# 2-[(*E*)-(6-Amino-1,3-dimethyl-2,4-dioxo-1,2,3,4-tetra­hydro­pyrimidin-5-yl)imino­meth­yl]pyridinium chloride monohydrate

**DOI:** 10.1107/S1600536811033307

**Published:** 2011-08-27

**Authors:** Irvin Booysen, Muhammed Ismail, Thomas Gerber, Eric Hosten, Richard Betz

**Affiliations:** a University of Kwazulu-Natal, School of Chemistry, Private Bag X01, Scottsville 3209, Pietermaritzburg, South Africa; b Nelson Mandela Metropolitan University, Summerstrand Campus, Department of Chemistry, University Way, Summerstrand, PO Box 77000, Port Elizabeth 6031, South Africa

## Abstract

The title compound, C_12_H_14_N_5_O_2_^+^·Cl^−^·H_2_O, is the monohydrate of the hydro­chloride of an oxopurine-derived Schiff base in which protonation took place at the pyridine N atom. The organic cation is essentially planar (r.m.s. of all fitted non-H atoms = 0.0373 Å). In the crystal, N—H⋯O and N—H⋯Cl hydrogen bonds as well as C—H⋯O and C—H⋯Cl contacts connect the different entities into a three-dimensional network. The shortest centroid–centroid distance between two pyrimidine rings is 3.6364 (9) Å.

## Related literature

For the development of radiopharmaceuticals, see: Gerber *et al.* (2011 ▶). For the crystal structure of the neutral organic parent ligand, see: Booysen *et al.* (2011*a* ▶). For the crystal structures of polymorphs of 6-amino-1,3-dimethyl-5-[(*E*-2-(methyl­sulfan­yl)benzyl­idene­amino]pyrimidine-2,4(1*H*,3*H*)-dione, see: Booy­sen *et al.* (2011*b* ▶,*c* ▶). For graph-set analysis of hydrogen bonds, see: Etter *et al.* (1990 ▶); Bernstein *et al.* (1995 ▶). For puckering analysis, see: Cremer & Pople (1975 ▶).
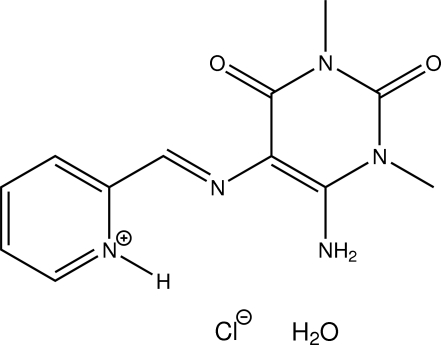


## Experimental

### 

#### Crystal data

C_12_H_14_N_5_O_2_^+^·Cl^−^·H_2_O
*M* *_r_* = 313.75
Orthorhombic, 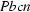

*a* = 13.3797 (4) Å
*b* = 15.7975 (5) Å
*c* = 12.9998 (4) Å
*V* = 2747.71 (15) Å^3^
*Z* = 8
Mo *K*α radiation
μ = 0.30 mm^−1^
*T* = 200 K
0.49 × 0.09 × 0.08 mm

#### Data collection

Bruker APEXII CCD diffractometer
Absorption correction: multi-scan (*SADABS*; Bruker, 2008 ▶) *T*_min_ = 0.879, *T*_max_ = 1.000
24436 measured reflections
3415 independent reflections
2327 reflections with *I* > 2σ(*I*)
*R*_int_ = 0.053

#### Refinement

*R*[*F* ^2^ > 2σ(*F* ^2^)] = 0.042
*wR*(*F* ^2^) = 0.111
*S* = 1.03
3415 reflections
212 parameters
H atoms treated by a mixture of independent and constrained refinement
Δρ_max_ = 0.34 e Å^−3^
Δρ_min_ = −0.22 e Å^−3^

### 

Data collection: *APEX2* (Bruker, 2010 ▶); cell refinement: *SAINT* (Bruker, 2010 ▶); data reduction: *SAINT*; program(s) used to solve structure: *SIR97* (Altomare *et al.*, 1999 ▶); program(s) used to refine structure: *SHELXL97* (Sheldrick, 2008 ▶); molecular graphics: *ORTEP-3* (Farrugia, 1997 ▶) and *Mercury* (Macrae *et al.*, 2008 ▶); software used to prepare material for publication: *SHELXL97* and *PLATON* (Spek, 2009 ▶).

## Supplementary Material

Crystal structure: contains datablock(s) I, global. DOI: 10.1107/S1600536811033307/dn2714sup1.cif

Supplementary material file. DOI: 10.1107/S1600536811033307/dn2714Isup2.cdx

Structure factors: contains datablock(s) I. DOI: 10.1107/S1600536811033307/dn2714Isup3.hkl

Supplementary material file. DOI: 10.1107/S1600536811033307/dn2714Isup4.cml

Additional supplementary materials:  crystallographic information; 3D view; checkCIF report

## Figures and Tables

**Table 1:** Hydrogen-bond geometry (Å, °)

*D*—H⋯*A*	*D*—H	H⋯*A*	*D*⋯*A*	*D*—H⋯*A*
N4—H741⋯O90^i^	0.82 (2)	2.12 (2)	2.901 (2)	158 (2)
N4—H742⋯Cl1	0.94 (2)	2.29 (2)	3.1565 (16)	153 (2)
N5—H751⋯Cl1	0.90 (2)	2.19 (2)	3.0255 (16)	155 (2)
O90—H901⋯O1^ii^	0.89 (3)	2.01 (3)	2.874 (2)	164 (3)
O90—H902⋯Cl1	0.82 (3)	2.43 (3)	3.213 (2)	159 (3)
C5—H5*A*⋯Cl1^iii^	0.98	2.83	3.642 (2)	141
C9—H9⋯Cl1^iv^	0.95	2.71	3.5912 (19)	155
C10—H10⋯O1^v^	0.95	2.65	3.564 (2)	163
C12—H12⋯O2^vi^	0.95	2.37	3.294 (2)	164

Symmetry codes: (i) 
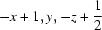
; (ii) 
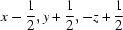
; (iii) 
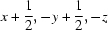
; (iv) 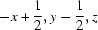; (v) 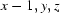; (vi) 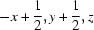.

**Fig. 1.**
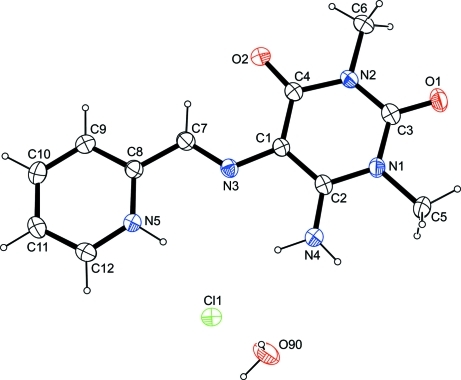
The molecular structure of the title compound, with atom labels and anisotropic displacement ellipsoids (drawn at 50% probability level).

**Fig. 2.**
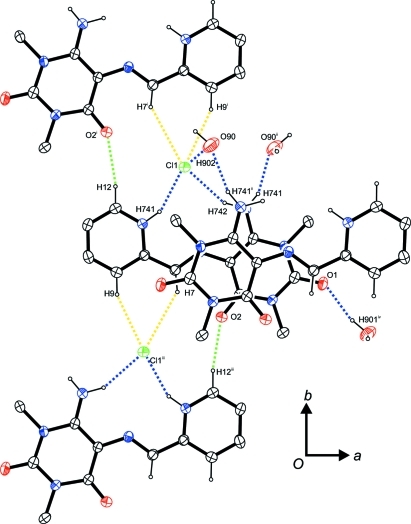
Intermolecular contacts, viewed along [0 0 -1]. For clarity, only hydrogen atoms participating in X—H···Y contacts are depicted. Blue dashed lines indicate classical hydrogen bonds, green dashed lines C–H···O contacts and yellow dashed lines C–H···Cl contacts. [Symmetry codes: (i) -x + 1/2, y + 1/2, z; (ii) -x + 1, y, -z + 1/2; (iii) -x + 1/2, y - 1/2, z; (iv) x + 1/2, y - 1/2, -z + 1/2].

**Fig. 3.**
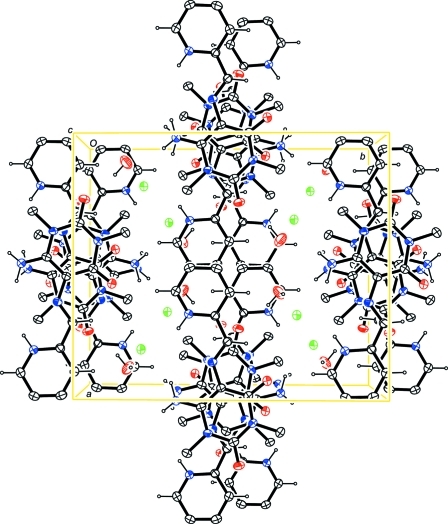
Molecular packing of the title compound, viewed along [0 0 -1] (anisotropic displacement ellipsoids drawn at 50% probability level). For clarity, only hydrogen atoms participating in X—H···Y contacts are depicted.

## supplementary crystallographic information

### Comment

Next to cardiovascular diseases, cancer has become one of the main fatal diseases in industrialized countries. Apart from classical surgery, chemo- and radiotherapeutic treatments have entered the arsenal of possible cures for certain types of cancer. All methods, however, suffer from their own set of problematic side-effects and, as a consequence, the development of radiopharmaceuticals – combining the advantages of chemotherapy as well as radiation methods while at the same time avoiding their unique respective undesired side-effects – has been a topic of research (Gerber *et al.*, 2011). Tailoring and fine-tuning of the envisioned radiopharmaceuticals' properties such as lipophilicity and, in particular, inertness is of paramount importance with respect to possible future *in vivo* applications in contemporary medicine and requires sound knowledge about structural parameters of the ligands applied if a more heuristic approach in the synthesis is to triumph over pure trial-and-error as it is encountered in this specific field of coordination chemistry up to the present day. To allow for an assessment of changes in structural features upon coordination has taken place, the molecular and crystal structure of the title compound has been determined. Information about metrical parameters of the neutral compound (Booysen *et al.*, 2011*a*) as well as other 6-amino-1,3-dimethyl-2,4(1*H*,3*H*)-dione-derived Schiff-base ligands (Booysen *et al.*, 2011*b*; Booysen *et al.*, 2011*c*) is apparent in the literature.

Protonation of the neutral organic ligand took place on the nitrogen atom of the pyridine moiety. The C=N bond is (*E*)-configured. Intracyclic angles in the protonated pyridine moiety cover a range of 117.73 (16)–123.66 (16) ° with the biggest angle on the protonated nitrogen atom and the smallest angle on the carbon atom bonded to the exocyclic substituent. The organic cation is essentially planar (r.m.s. for all its fitted non-hydrogen atoms = 0.0373 Å). The low puckering amplitude (τ = 2.2 °, r.m.s. for all its fitted and bonded non-hydrogen atoms = 0.0373 Å) of the non-aromatic heterocycle precludes a conformational analysis (Cremer & Pople, 1975) (Fig. 1).

In the crystal, hydrogen bonds as well as C–H···O and C–H···Cl contacs are observed whose range fall by about and more than 0.1 Å below the sum of van-der-Waals radii of the atoms participating for the latter two types of interaction. The classical hydrogen bonds are supported by the protons of the water molecule as well as the amino group and have the chloride ion, the oxygen atom of the water molecule and the oxygen atom of the keto group located between the two methylated nitrogen atoms as acceptor. Two C–H···O contacts can be observed: the first one stemming from the C–H group in *ortho*-position to the protonated nitrogen atom in the pyridyl moiety and the keto group not acting as acceptor for a classical hydrogen bond and the second one between the C–H group in *para* position to the protonated nitrogen atom of the pyridyl moiety and the keto group that is already acting as acceptor for one of the classical hydrogen bonds. The two C–H···Cl contacts apply the vinylic hydrogen atom as well as one of the C–H groups present in the pyridyl moiety as donors. The chloride anion thus is pentacoordinate. A description of the classical hydrogen bonds in terms of graph-set analysis (Etter *et al.*, 1990; Bernstein *et al.*, 1995) necessitates a *DDDDD* descriptor on the unitary level while the C–H···O as well as the C–H···Cl contacts can be described by means of a *DDC*^1^_1_(9)*C*^1^_1_(11) descriptor on the same level. In total, the entities of the title compound are connected to a three-dimensional network. The shortest intercentroid distance between two centers of gravity was found at 3.6364 (9) Å (Fig. 2).

The packing of the title compound in the crystal structure is shown in Figure 3.

### Experimental

The title compound was prepared by the reaction of (*E*)-6-amino-1,3- dimethyl-5-(pyridin-2-ylmethyleneamino)pyrimidine-2,4(1*H*,3*H*)- dione and *trans*-[ReOCl_3_(PPh_3_)_2_] in acetonitrile. The solution was filtered and single crystals suitable for the X-ray analysis were obtained from the mother liquor which was left in a fridge over several days.

### Refinement

Carbon-bound H atoms were placed in calculated positions (C—H 0.95 Å) and were included in the refinement in the riding model approximation, with *U*(H) set to 1.2*U*_eq_(C). The H atoms of the methyl groups were allowed to rotate with a fixed angle around the C—C bond to best fit the experimental electron density (HFIX 137 in the *SHELX* program suite (Sheldrick, 2008)), with *U*(H) set to 1.5*U*_eq_(C). All nitrogen-bound H atoms as well as the hydrogen atoms of the molecule of crystal water were located on a difference Fourier map and refine freely.

### Crystal data

**Table:**

C_12_H_14_N_5_O_2_^+^·Cl^−^·H_2_O	*F*(000) = 1312
*M**_r_* = 313.75	*D*_x_ = 1.517 Mg m^−^^3^
Orthorhombic, *P**b**c**n*	Mo *K*α radiation, λ = 0.71073 Å
Hall symbol: -P 2n 2ab	Cell parameters from 5575 reflections
*a* = 13.3797 (4) Å	θ = 2.5–28.1°
*b* = 15.7975 (5) Å	µ = 0.30 mm^−^^1^
*c* = 12.9998 (4) Å	*T* = 200 K
*V* = 2747.71 (15) Å^3^	Needle, brown
*Z* = 8	0.49 × 0.09 × 0.08 mm

### Data collection

**Table:**

Bruker APEXII CCD diffractometer	3415 independent reflections
Radiation source: fine-focus sealed tube	2327 reflections with *I* > 2σ(*I*)
graphite	*R*_int_ = 0.053
φ and ω scans	θ_max_ = 28.3°, θ_min_ = 2.0°
Absorption correction: multi-scan (*SADABS*; Bruker, 2008)	*h* = −17→17
*T*_min_ = 0.879, *T*_max_ = 1.000	*k* = −21→20
24436 measured reflections	*l* = −17→12

### Refinement

**Table:**

Refinement on *F*^2^	Primary atom site location: structure-invariant direct methods
Least-squares matrix: full	Secondary atom site location: difference Fourier map
*R*[*F*^2^ > 2σ(*F*^2^)] = 0.042	Hydrogen site location: inferred from neighbouring sites
*wR*(*F*^2^) = 0.111	H atoms treated by a mixture of independent and constrained refinement
*S* = 1.03	*w* = 1/[σ^2^(*F*_o_^2^) + (0.0549*P*)^2^ + 0.7712*P*] where *P* = (*F*_o_^2^ + 2*F*_c_^2^)/3
3415 reflections	(Δ/σ)_max_ < 0.001
212 parameters	Δρ_max_ = 0.34 e Å^−^^3^
0 restraints	Δρ_min_ = −0.22 e Å^−^^3^

### Fractional atomic coordinates and isotropic or equivalent isotropic displacement parameters (Å^2^)

**Table:**

	*x*	*y*	*z*	*U*_iso_*/*U*_eq_	
O1	0.75612 (9)	−0.02776 (9)	0.13036 (10)	0.0310 (3)	
O2	0.43377 (9)	−0.11801 (8)	0.12425 (10)	0.0282 (3)	
N1	0.63382 (10)	0.07221 (9)	0.12603 (11)	0.0222 (3)	
N2	0.59510 (11)	−0.07228 (9)	0.13227 (11)	0.0224 (3)	
N3	0.36366 (10)	0.05754 (9)	0.12000 (11)	0.0201 (3)	
N4	0.51074 (12)	0.17487 (10)	0.11875 (13)	0.0260 (3)	
H741	0.5501 (17)	0.2148 (14)	0.1230 (14)	0.026 (5)*	
H742	0.4432 (19)	0.1906 (15)	0.1194 (16)	0.040 (6)*	
N5	0.18069 (11)	0.13375 (9)	0.11007 (12)	0.0246 (3)	
H751	0.2352 (17)	0.1666 (15)	0.1123 (15)	0.034 (6)*	
C1	0.46118 (12)	0.02987 (11)	0.12260 (12)	0.0194 (3)	
C2	0.53451 (12)	0.09375 (11)	0.12252 (13)	0.0202 (3)	
C3	0.66668 (13)	−0.01110 (11)	0.12946 (13)	0.0224 (4)	
C4	0.49100 (13)	−0.05750 (11)	0.12589 (13)	0.0205 (3)	
C5	0.71013 (14)	0.13949 (12)	0.12604 (18)	0.0344 (5)	
H5A	0.7050	0.1723	0.0623	0.052*	
H5B	0.7767	0.1140	0.1307	0.052*	
H5C	0.6994	0.1769	0.1851	0.052*	
C6	0.62944 (15)	−0.16046 (12)	0.13966 (17)	0.0329 (5)	
H6A	0.6593	−0.1777	0.0741	0.049*	
H6B	0.5725	−0.1973	0.1554	0.049*	
H6C	0.6794	−0.1652	0.1945	0.049*	
C7	0.28582 (12)	0.00923 (11)	0.12190 (13)	0.0230 (4)	
H7	0.2925	−0.0506	0.1257	0.028*	
C8	0.18734 (13)	0.04871 (11)	0.11802 (13)	0.0221 (4)	
C9	0.09840 (14)	0.00262 (12)	0.12149 (15)	0.0281 (4)	
H9	0.1001	−0.0572	0.1288	0.034*	
C10	0.00810 (15)	0.04423 (13)	0.11423 (16)	0.0332 (5)	
H10	−0.0525	0.0130	0.1171	0.040*	
C11	0.00552 (15)	0.13148 (13)	0.10267 (17)	0.0368 (5)	
H11	−0.0564	0.1603	0.0951	0.044*	
C12	0.09408 (14)	0.17558 (12)	0.10235 (16)	0.0315 (5)	
H12	0.0938	0.2355	0.0967	0.038*	
Cl1	0.31571 (3)	0.28729 (3)	0.11637 (4)	0.03282 (15)	
O90	0.39829 (14)	0.33807 (11)	0.34015 (17)	0.0472 (4)	
H901	0.350 (2)	0.373 (2)	0.359 (2)	0.063 (8)*	
H902	0.393 (2)	0.326 (2)	0.279 (3)	0.082 (12)*	

### Atomic displacement parameters (Å^2^)

**Table:**

	*U*^11^	*U*^22^	*U*^33^	*U*^12^	*U*^13^	*U*^23^
O1	0.0182 (6)	0.0340 (7)	0.0406 (8)	0.0043 (5)	0.0002 (6)	0.0034 (7)
O2	0.0245 (7)	0.0204 (6)	0.0397 (8)	−0.0011 (5)	0.0006 (6)	−0.0014 (6)
N1	0.0162 (7)	0.0237 (7)	0.0267 (8)	−0.0009 (6)	0.0007 (6)	0.0023 (7)
N2	0.0199 (7)	0.0202 (7)	0.0271 (8)	0.0037 (6)	0.0004 (6)	0.0006 (6)
N3	0.0189 (7)	0.0230 (7)	0.0184 (7)	0.0015 (6)	0.0006 (6)	−0.0013 (6)
N4	0.0205 (8)	0.0209 (7)	0.0366 (9)	−0.0011 (6)	0.0005 (7)	0.0001 (7)
N5	0.0193 (7)	0.0204 (7)	0.0342 (9)	−0.0008 (6)	−0.0009 (7)	−0.0007 (7)
C1	0.0183 (8)	0.0210 (8)	0.0187 (8)	0.0012 (6)	−0.0001 (7)	0.0005 (7)
C2	0.0185 (8)	0.0250 (8)	0.0170 (8)	0.0026 (7)	0.0005 (7)	0.0010 (7)
C3	0.0219 (8)	0.0280 (9)	0.0174 (9)	0.0035 (7)	0.0005 (7)	0.0019 (8)
C4	0.0207 (8)	0.0224 (8)	0.0183 (8)	0.0021 (7)	0.0002 (7)	−0.0001 (7)
C5	0.0192 (9)	0.0264 (10)	0.0575 (14)	−0.0036 (7)	0.0026 (9)	0.0057 (10)
C6	0.0263 (9)	0.0220 (9)	0.0505 (13)	0.0058 (7)	−0.0019 (9)	−0.0013 (9)
C7	0.0218 (8)	0.0227 (8)	0.0244 (9)	0.0020 (7)	−0.0001 (8)	−0.0010 (7)
C8	0.0213 (8)	0.0220 (8)	0.0229 (9)	0.0010 (7)	−0.0018 (7)	−0.0020 (7)
C9	0.0252 (9)	0.0220 (8)	0.0370 (11)	−0.0016 (7)	−0.0011 (9)	−0.0005 (8)
C10	0.0210 (9)	0.0332 (10)	0.0455 (12)	−0.0050 (8)	0.0005 (9)	−0.0027 (10)
C11	0.0202 (9)	0.0298 (10)	0.0603 (15)	0.0047 (8)	−0.0007 (9)	−0.0005 (10)
C12	0.0260 (9)	0.0218 (9)	0.0467 (13)	0.0033 (8)	0.0011 (9)	−0.0006 (8)
Cl1	0.0254 (2)	0.0196 (2)	0.0535 (3)	0.00017 (17)	−0.0032 (2)	−0.0015 (2)
O90	0.0466 (10)	0.0376 (9)	0.0575 (12)	0.0172 (8)	−0.0031 (9)	−0.0016 (8)

### Geometric parameters (Å, °)

**Table:**

O1—C3	1.225 (2)	C5—H5A	0.9800
O2—C4	1.225 (2)	C5—H5B	0.9800
N1—C2	1.372 (2)	C5—H5C	0.9800
N1—C3	1.388 (2)	C6—H6A	0.9800
N1—C5	1.474 (2)	C6—H6B	0.9800
N2—C3	1.361 (2)	C6—H6C	0.9800
N2—C4	1.415 (2)	C7—C8	1.459 (2)
N2—C6	1.470 (2)	C7—H7	0.9500
N3—C7	1.291 (2)	C8—C9	1.396 (2)
N3—C1	1.377 (2)	C9—C10	1.379 (3)
N4—C2	1.321 (2)	C9—H9	0.9500
N4—H741	0.82 (2)	C10—C11	1.387 (3)
N4—H742	0.94 (2)	C10—H10	0.9500
N5—C12	1.338 (2)	C11—C12	1.374 (3)
N5—C8	1.350 (2)	C11—H11	0.9500
N5—H751	0.90 (2)	C12—H12	0.9500
C1—C2	1.407 (2)	O90—H901	0.89 (3)
C1—C4	1.437 (2)	O90—H902	0.82 (3)
			
C2—N1—C3	122.87 (15)	N1—C5—H5C	109.5
C2—N1—C5	119.47 (15)	H5A—C5—H5C	109.5
C3—N1—C5	117.66 (14)	H5B—C5—H5C	109.5
C3—N2—C4	125.03 (14)	N2—C6—H6A	109.5
C3—N2—C6	117.05 (14)	N2—C6—H6B	109.5
C4—N2—C6	117.90 (14)	H6A—C6—H6B	109.5
C7—N3—C1	125.19 (15)	N2—C6—H6C	109.5
C2—N4—H741	125.9 (15)	H6A—C6—H6C	109.5
C2—N4—H742	119.3 (14)	H6B—C6—H6C	109.5
H741—N4—H742	114 (2)	N3—C7—C8	118.36 (16)
C12—N5—C8	123.66 (16)	N3—C7—H7	120.8
C12—N5—H751	114.9 (14)	C8—C7—H7	120.8
C8—N5—H751	121.4 (14)	N5—C8—C9	117.73 (16)
N3—C1—C2	115.66 (15)	N5—C8—C7	119.18 (16)
N3—C1—C4	124.67 (15)	C9—C8—C7	123.09 (16)
C2—C1—C4	119.67 (15)	C10—C9—C8	119.75 (17)
N4—C2—N1	118.34 (16)	C10—C9—H9	120.1
N4—C2—C1	121.85 (15)	C8—C9—H9	120.1
N1—C2—C1	119.81 (15)	C9—C10—C11	120.20 (18)
O1—C3—N2	122.31 (16)	C9—C10—H10	119.9
O1—C3—N1	120.87 (16)	C11—C10—H10	119.9
N2—C3—N1	116.82 (15)	C12—C11—C10	118.85 (18)
O2—C4—N2	119.19 (15)	C12—C11—H11	120.6
O2—C4—C1	125.12 (16)	C10—C11—H11	120.6
N2—C4—C1	115.68 (15)	N5—C12—C11	119.75 (18)
N1—C5—H5A	109.5	N5—C12—H12	120.1
N1—C5—H5B	109.5	C11—C12—H12	120.1
H5A—C5—H5B	109.5	H901—O90—H902	111 (3)
			
C7—N3—C1—C2	−178.39 (16)	C6—N2—C4—O2	−1.7 (3)
C7—N3—C1—C4	1.2 (3)	C3—N2—C4—C1	−4.0 (3)
C3—N1—C2—N4	−179.57 (16)	C6—N2—C4—C1	177.82 (16)
C5—N1—C2—N4	0.3 (3)	N3—C1—C4—O2	1.9 (3)
C3—N1—C2—C1	0.2 (3)	C2—C1—C4—O2	−178.50 (17)
C5—N1—C2—C1	−179.93 (17)	N3—C1—C4—N2	−177.63 (15)
N3—C1—C2—N4	−0.9 (2)	C2—C1—C4—N2	2.0 (2)
C4—C1—C2—N4	179.47 (17)	C1—N3—C7—C8	−179.65 (15)
N3—C1—C2—N1	179.36 (15)	C12—N5—C8—C9	−2.0 (3)
C4—C1—C2—N1	−0.3 (2)	C12—N5—C8—C7	177.56 (18)
C4—N2—C3—O1	−176.43 (17)	N3—C7—C8—N5	1.6 (3)
C6—N2—C3—O1	1.8 (3)	N3—C7—C8—C9	−178.81 (17)
C4—N2—C3—N1	3.9 (3)	N5—C8—C9—C10	1.6 (3)
C6—N2—C3—N1	−177.86 (16)	C7—C8—C9—C10	−177.98 (18)
C2—N1—C3—O1	178.46 (16)	C8—C9—C10—C11	0.5 (3)
C5—N1—C3—O1	−1.4 (3)	C9—C10—C11—C12	−2.3 (3)
C2—N1—C3—N2	−1.9 (3)	C8—N5—C12—C11	0.3 (3)
C5—N1—C3—N2	178.23 (16)	C10—C11—C12—N5	1.9 (3)
C3—N2—C4—O2	176.49 (17)		

### Hydrogen-bond geometry (Å, °)

**Table:**

*D*—H···*A*	*D*—H	H···*A*	*D*···*A*	*D*—H···*A*
N4—H741···O90^i^	0.82 (2)	2.12 (2)	2.901 (2)	158 (2)
N4—H742···Cl1	0.94 (2)	2.29 (2)	3.1565 (16)	153 (2)
N5—H751···Cl1	0.90 (2)	2.19 (2)	3.0255 (16)	155 (2)
O90—H901···O1^ii^	0.89 (3)	2.01 (3)	2.874 (2)	164 (3)
O90—H902···Cl1	0.82 (3)	2.43 (3)	3.213 (2)	159 (3)
C5—H5A···Cl1^iii^	0.98	2.83	3.642 (2)	141.
C9—H9···Cl1^iv^	0.95	2.71	3.5912 (19)	155.
C10—H10···O1^v^	0.95	2.65	3.564 (2)	163.
C12—H12···O2^vi^	0.95	2.37	3.294 (2)	164.

Symmetry codes: (i) −*x*+1, *y*, −*z*+1/2; (ii) *x*−1/2, *y*+1/2, −*z*+1/2; (iii) *x*+1/2, −*y*+1/2, −*z*; (iv) −*x*+1/2, *y*−1/2, *z*; (v) *x*−1, *y*, *z*; (vi) −*x*+1/2, *y*+1/2, *z*.
